# Retrocecal Ascending Appendix Attached to the Hepatic Flexure and Right Intra-abdominal Testis Identified During Open Appendicectomy: A Case Report

**DOI:** 10.7759/cureus.57484

**Published:** 2024-04-02

**Authors:** Malik Amna Khatoon, Sarosh Naeem, Urooj Akmal, Ahsan Farid, Salman Ahmed Khan

**Affiliations:** 1 Orthopaedic Surgery, Dow University of Health Sciences, Dow International Medical College, Karachi, PAK; 2 General Surgery, Sindh Government Qatar Hospital, Karachi, PAK; 3 Internal Medicine, Dow International Medical College, Karachi, PAK

**Keywords:** intrabdominal testes, orchidectomy, intraabdominal appendix locations, crytochidism, retrocecal appendix

## Abstract

Appendicectomy, or the removal of the appendix, is an emergency procedure following symptomatic acute appendicitis. Diagnosis is made on clinical examination but can be confirmed on imaging if other abnormalities are suspected. A few variants of appendix anatomical position exist that can be difficult to manage. In addition, secondary findings during surgery can come unexpectedly. We report a case of a 14-year-old male, who presented to the emergency department at our government institution with abdominal pain and vomiting. Examination revealed an empty right scrotum, which was unnoticed by the patient and never examined previously due to residence in an area of limited healthcare access. Ultrasound done elsewhere was inconclusive. The surgical intervention showed a retrocecal appendix attached to an ascending colon terminating at hepatic flexure. The procedure was further complicated by the presence of the right intra-abdominal testis located below the cecum. Excised samples were sent for histopathology, and the patient was followed with biopsy reports. This case highlights the challenges encountered during routine appendicectomy with unusual findings.

## Introduction

Acute appendicitis (AA) is a common surgical emergency characterized by abdominal pain, fever, and anorexia. There are several positions in which the appendix is located according to its anatomical location such as retrocecal being the most common, followed by sub-cecal, pre-ileal, post-ileal, and pelvic [1]. Management and definitive treatment of AA is commonly via surgery upon confirming diagnosis with Alvarado scores [1]. Alvarado scoring consists of 10 points total based on six clinical findings and two laboratory markers [2]. A score of seven or more usually warrants surgical intervention, but further studies are needed to identify complicated cases of appendicitis that are not always straightforward even on clinical presentation [2].

Diagnosing and managing the condition can be challenging when it presents with atypical signs and symptoms. A retrocecal ascending appendix is a rare variant of the appendix's anatomical position in which the appendix is located behind the cecum and ascends upwards along the posterior abdominal wall towards the right upper quadrant [3]. Retrocecal appendicitis can form an abscess in the pararenal space and even spread to a bare area of the liver, causing an increase in complication rates if not caught early [4].

Several conditions can imitate appendicitis with differentials such as mesenteric adenitis, Crohn’s disease, renal colic, or pelvic inflammatory disease [1]. Specific to young males, other diseases reported in tangent to AA include undescended testes due to seminoma or perforated appendix, leading to scrotal abscess [5,6]. Abnormal findings during routine surgery are not uncommon but can take surgeons by surprise. For this reason, diagnostic imaging such as an ultrasound or computed tomography (CT) is crucial in difficult-to-diagnose cases, especially for children and non-obese young adults [7].

## Case presentation

A 14-year-old male of average height and build, residing in a remote village, presented to the emergency department with the complaint of right lower abdominal pain and vomiting with a one-day duration. The patient had an atypical presentation of epigastric pain that radiated to the right lumbar region and ultimately settled in the right iliac fossa. The patient had three episodes of non-bilious vomiting and no current or past history of associated medical illness or surgical intervention.

**Figure 1:**
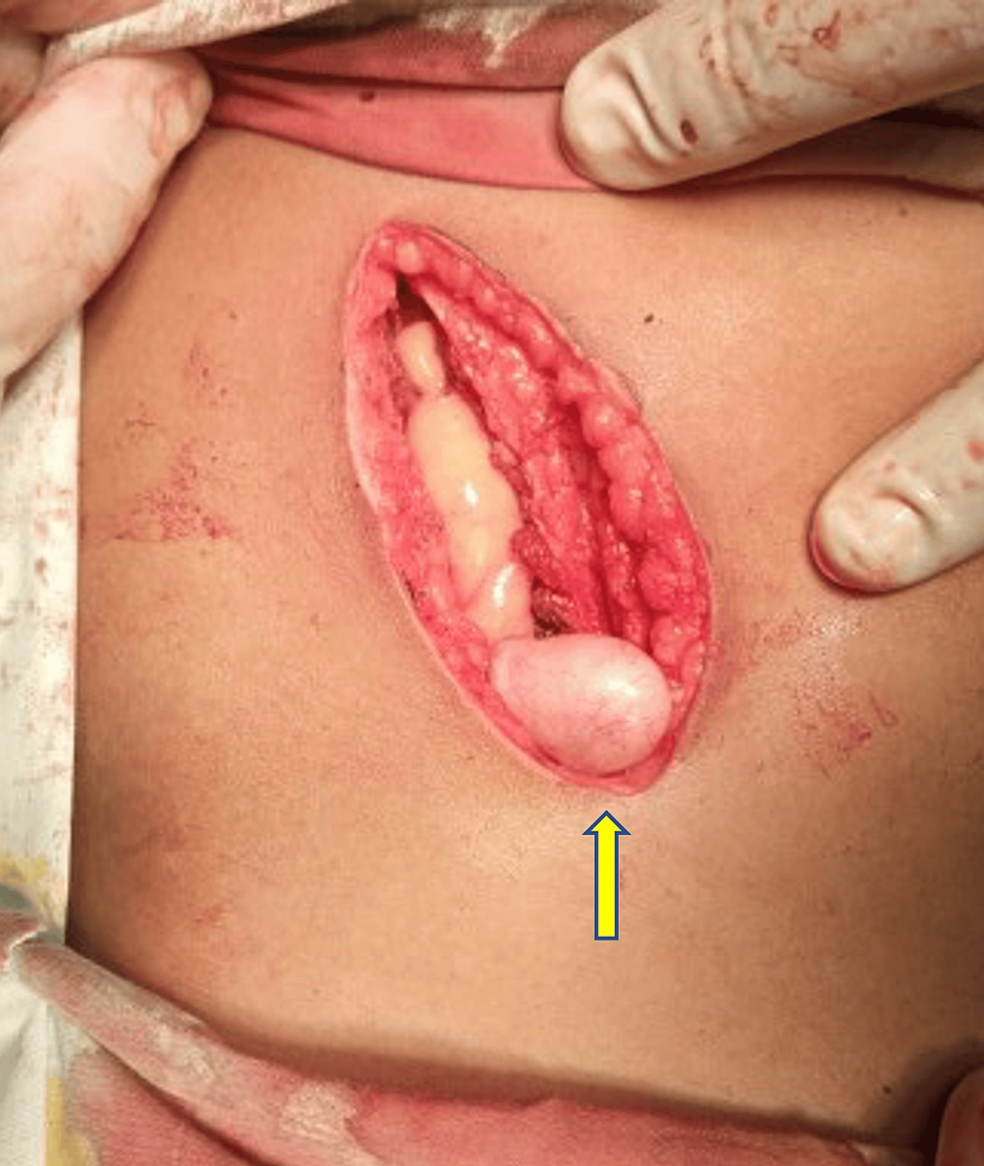
McBurney’s incision, right testis (yellow arrow).

On examination, tenderness was appreciated in the right lumbar region, rebound tenderness in the right iliac fossa, and positive psoas sign. Inguinoscrotal examination revealed an incidental empty right scrotum with an impalpable right testis, which was never noticed by the patient or examined in their medical history. The patient had an elevated leucocyte count of 14,000/mm^3^ and a calculated Alvarado score of eight out of ten, supporting the clinical diagnosis of acute appendicitis and an additional diagnosis of right undescended testes. A prior ultrasound done elsewhere showed ambiguous findings and a repeat ultrasound or additional imaging could not be performed because of the unavailability of the service at our institution at the time of patient presentation. Emergency informed consent was obtained for open appendicectomy and right orchidopexy/orchidectomy. During surgery, a McBurney's incision was made, revealing a retrocecal appendix curling behind the ascending colon with no tip in sight. The right testis, small with a short spermatic cord, was situated behind the cecum and brought forward (Figure 1).

**Figure 2:**
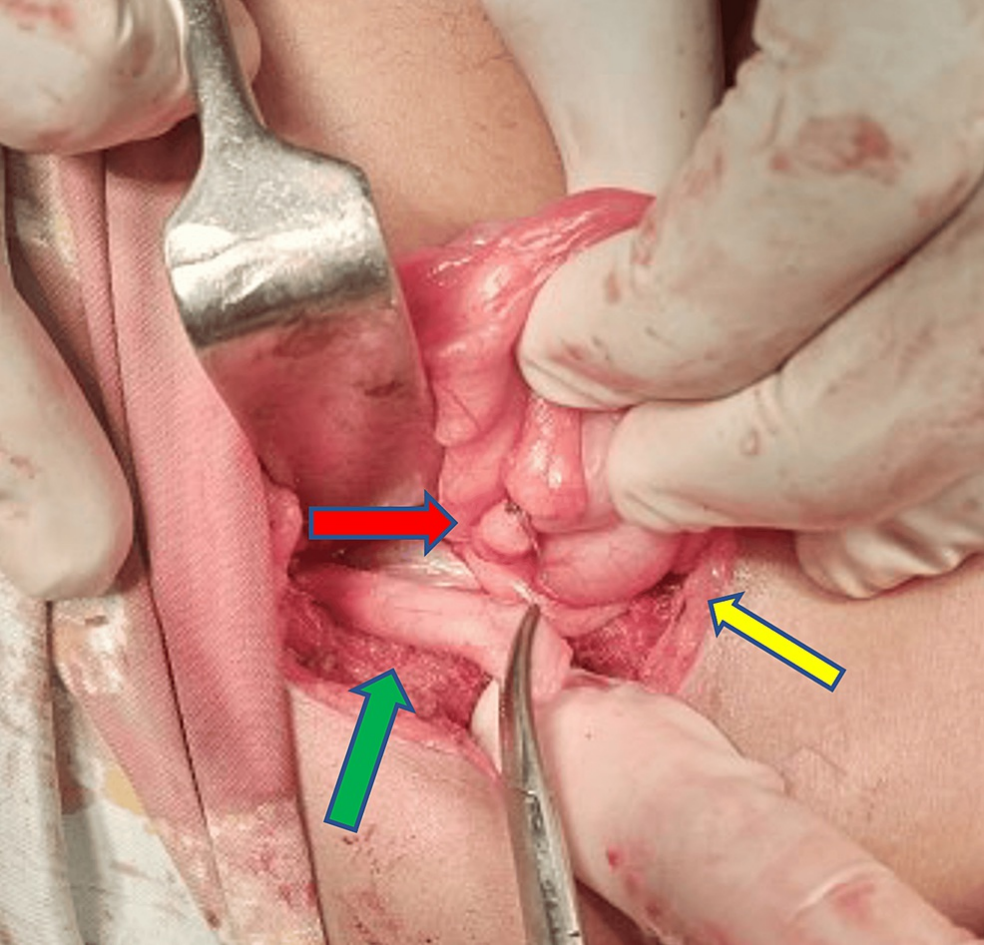
Base of appendix tied (red arrow), remaining appendiceal tissue (green arrow), right testis (yellow arrow).

After tying and resecting the appendiceal base, the incision was extended, and the rest of the appendix was followed through a retrograde approach (Figure 2).

**Figure 3:**
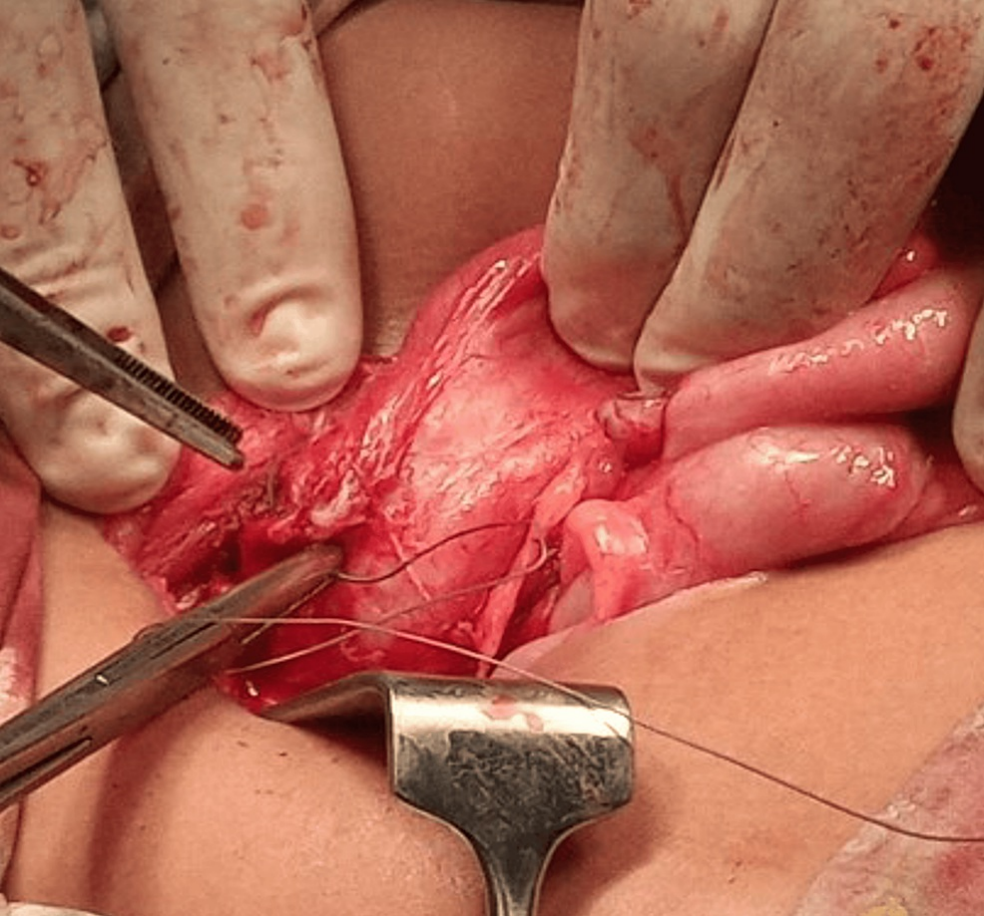
Appendiceal tissue joined with the ascending colon. Careful dissection is performed while transfixing bleeding vessels.

The ascending colon was mobilized, and careful dissection was done as the surface of the appendix was connected to the wall of the ascending colon (Figure 3).

**Figure 4:**
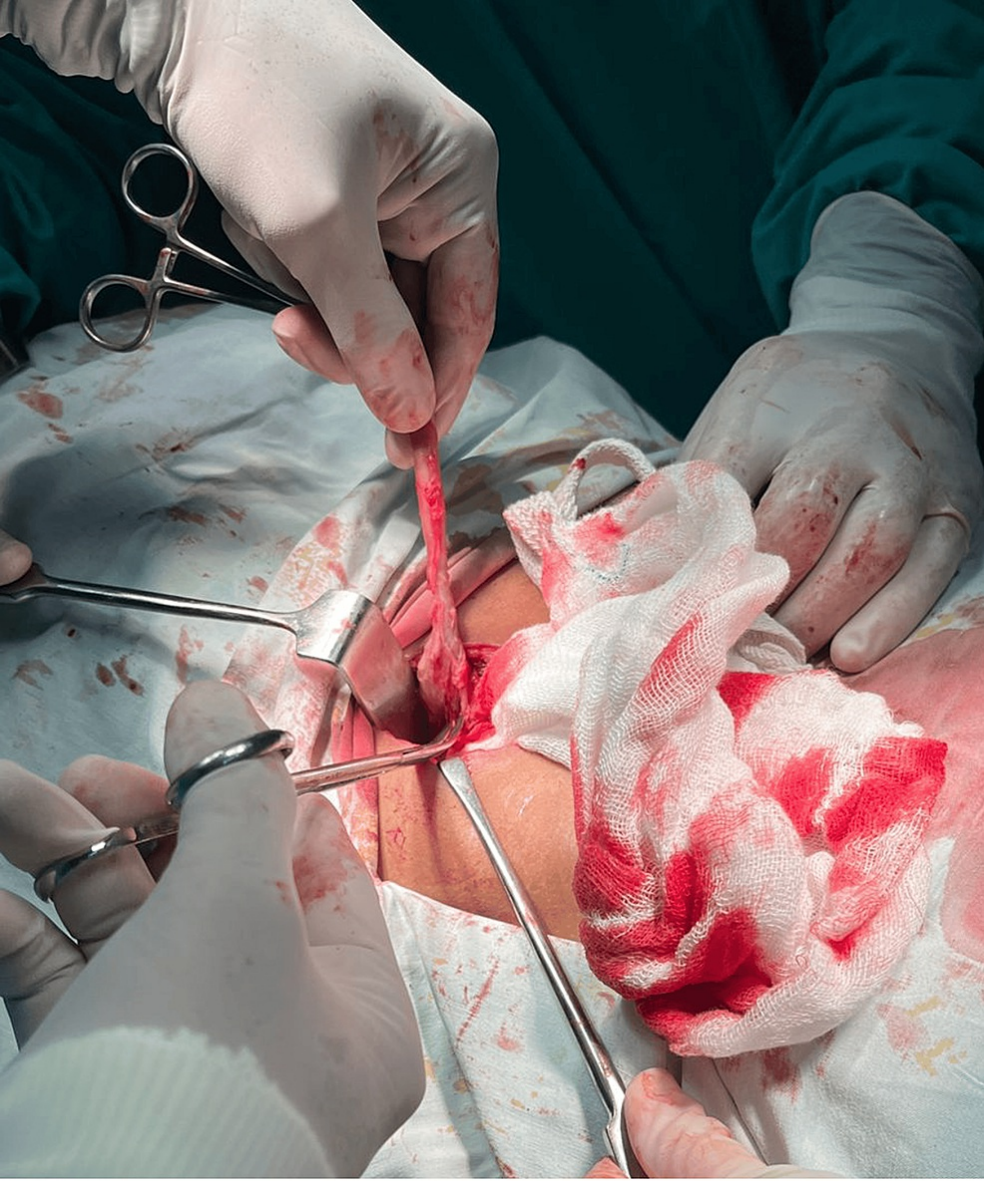
Appendiceal tissue dissected from ascending colon.

An inflamed lump at the tip was found attached to the hepatic flexure (Figure 4).

**Figure 5:**
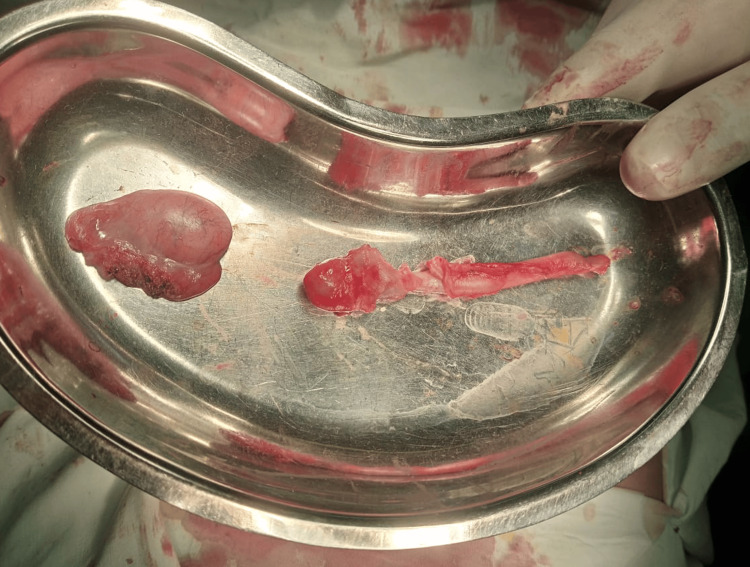
Excised right testis and appendix with an inflamed lump at the tip.

Orchidectomy was performed because of the small nature of the right testis, short cord, and inability to reach the deep inguinal ring. The excised appendix and testis were sent for histopathology (Figure 5).

After an uneventful postoperative period, the patient was discharged on prophylactic oral antibiotics and followed for biopsy reports. The histopathology reports confirmed an acute suppurative appendicitis with necrotic lumen and atrophic testis (2.5 cm x 2 cm x 1 cm) with interstitial fibrosis and no evidence of malignancy.

## Discussion

Even though clinical assessment remains the mainstay for the diagnosis of AA, imaging can be a reliable source to rule out exclusions or unconventional diagnoses in cases of atypical presentations [7]. The Alvarado score, the first developed scoring system for diagnosing AA, is widely accepted, but many institutions even within the same country still differ on their method for confirming AA in a patient [7]. The reason for this could be the location of the institution and the availability of imaging. Our patient presented from a remote village with no healthcare facilities nearby. Even government institutions in major cities of Pakistan have insufficient availability of imaging options or professional personnel trained to give their expert opinion [8].

Limited access to healthcare in certain countries and expenses of advanced imaging modalities can be a factor in the accurate diagnosis of this disease. Alvarado scoring has been a simple and affordable tool in emergency settings as surgery treatment is a definitive and safe option for appendicitis [2]. Our government institution relies on clinical diagnosis, especially for patients presenting in the emergency department. In these situations, risking a delay in treatment can cause life-threatening complications. With the risk of perforation or further deterioration, most surgeons opt for early surgical intervention in cases of AA and accept the negative appendicectomy rate of 15-20% [9].

Retrocecal appendicitis may mimic symptoms of gastric or intestinal pathologies, but its extension to the liver or other surrounding organs can cause life-threatening perforation [4]. A point-of-care ultrasound (POCUS) has been beneficial in cases of retrocecal appendicitis. If available, an experienced POCUS practitioner scans the right upper to lower quadrant of the abdomen and can expedite diagnosis in patients with late presentation or those with suspected perforation [10]. This can avoid the potential need for invasive modalities such as CT scans.

Undescended testes (UDT), or cryptorchidism, is a common congenital disorder that is divided into palpable or non-palpable testes (NPT) [11]. It is also a risk factor for future malignancy, infertility, and other adverse outcomes [12]. Our patient presented with NPT and had no prior knowledge of his condition. Again, having never been to a physician for examination, our patient presented late with this disorder, which warranted the excision of the testis. If detected early, surgical intervention by the age of 18 months can prevent testicular degenerative changes and reduce the chances of conversion to malignancy [11].

Intra-abdominal testes (IAT) are located in 30% of NPT and require laparoscopic techniques for accurate diagnosis [12]. Testicular position and laxity of the spermatic cord are factors to consider when the surgeon decides ultimate outcome for IAT [12]. A normal testis size in post-pubertal males is 4-5 cm length x 2.5 cm width and 3 cm height; on gross examination, small testes are those smaller than the 50th percentile for age or 20% smaller than the contra-lateral testis on physical examination [13]. In the case of our patient, the spermatic cord length was not adequate to reach the scrotum, and the testes were grossly small in appearance, so an appropriate decision was taken to excise and send for a biopsy.

Incidental findings during abdominal surgeries are common, and more information on this matter can help surgeons deal with the unexpected [14]. Ultimately retrocecal ascending appendicitis should be considered when appendicitis symptoms are atypical and ultrasound findings do not support another diagnosis [4]. Treatment for cryptorchidism should be initiated maximum by six months of age if the testes have not descended in the scrotal region [11]. More reporting on unusual discoveries during routine procedures, such as epidemiological data on incidental findings and techniques or steps followed, can help surgeons anticipate adverse outcomes and deal with them accordingly [14].

We have to mention again that we are a small government institution with limited resources. Access to costly laboratory or radiological tests can be a burden and out of reach for many patients as they present from rural areas. However, only patients who are deemed by senior consultants to operate or manage within our limits are admitted, and those needing advanced treatment are referred to tertiary care centers. The limitation of our case report was the unavailability of imaging with a diagnostic professional and laparoscope at the time of patient presentation, which could have detected the atypical position of the appendix and IAT.

## Conclusions

AA is a surgical emergency and intervention is needed in a timely manner to avoid delays and complications. Unusual findings should be anticipated in atypical cases and settings of limited imaging and diagnostic access. The best decision should be taken bearing in mind the circumstances of the patient and availability of imaging and expertise. To our knowledge, this is the first reported case of retrocecal ascending appendix attached to hepatic flexure and the right undescended testis situated behind the cecum in a young male patient.
